# Delineation of Novel Compound Heterozygous Variants in LTBP2 Associated with Juvenile Open Angle Glaucoma

**DOI:** 10.3390/genes9110527

**Published:** 2018-10-30

**Authors:** Osamah Saeedi, Sairah Yousaf, Joby Tsai, Kathleen Palmer, Saima Riazuddin, Zubair M. Ahmed

**Affiliations:** 1 Department of Ophthalmology and Visual Sciences, University of Maryland School of Medicine, Baltimore, MD 21201, USA; jojoby210@gmail.com; 2 Department of Otorhinolaryngology Head and Neck Surgery, University of Maryland School of Medicine, Baltimore, MD 21201, USA; syousaf@som.umaryland.edu (S.Y.); sriazuddin@som.umaryland.edu (S.R.); 3 Program of Personalized and Genomic Medicine, University of Maryland School of Medicine, Baltimore, MD 21201, USA; kpalmer@som.umaryland.edu

**Keywords:** glaucoma, juvenile-onset open angle glaucoma, JOAG, *LTBP2*, optic neuropathy, compound heterozygous

## Abstract

Juvenile open angle glaucoma (JOAG), which is an uncommon form of primary open angle glaucoma, is a clinically and genetically heterogeneous disorder. We report on a family with a recessively inherited form of JOAG. The proband has a superior and an inferior never fiber layer thinning in both the eyes and the nasal visual field (VF) defects in the left eye, which are clinical findings consistent with glaucomatous optic neuropathy. Whole exome sequencing revealed two novel compound heterozygous variants [c.2966C>G, p.(Pro989Arg); c.5235T>G, p.(Asn1745Lys)] in latent transforming growth factor-beta-binding protein 2 (*LTBP2*) segregating with the phenotype. Both these variants are predicted to replace evolutionary conserved amino acids, have a pathogenic effect on the encode protein, and have very low frequencies in the control databases. Mutations in LTBP2 are known to cause the Weill-Marchesani syndrome and a Weill-Marchesani-like syndrome, which include glaucoma in their clinical presentation. However, to our knowledge, this is the first published case of a JOAG subject associated with recessively inherited variants of LTPB2 and, thus, expands the repertoire of the known genetic causes of JOAG and the phenotypic spectrum of LTBP2 alleles.

## 1. Introduction

Glaucoma affects about 66 million people worldwide and is the second leading cause of blindness worldwide [1]. This disease is a slowly progressing optic neuropathy associated with an elevated intraocular pressure (IOP) and results in damage to retinal ganglion cells and their axons [1]. Primary open angle glaucoma (POAG) affects about 3 million people in the United States and is the most common form of glaucoma worldwide [1,2]. Juvenile open angle glaucoma (JOAG), which is an uncommon form of POAG that is usually inherited in an autosomal dominant fashion, is defined by onset in young adulthood before 40 years of age [3,4]. JOAG patients typically present with a higher IOP and more severe optic nerve damage when compared to adult-onset POAG [5]. The prevalence of JOAG has been estimated to be between 4% of all childhood glaucoma and 0.038% of the general population [4,6,7]. There have been multiple genetic mutations associated with JOAG such as myocilin (*MYOC*), cytochrome P450, family 1, subfamily B (*CYP1B1*), and optineurin (*OPTN*) [5].

In this paper, we identified two novel heterozygous variants in the latent transforming growth factor-beta-binding protein 2 (LTPB2) associated with the JOAG. LTBP2 is an 1821-amino acid protein with a high expression in the Descemet’s membrane and in the lens capsule as well as in the non-pigmented epithelium of the ciliary processes, trabecular meshwork, and the transitional zone between the sclera and corneal stroma [8,9]. There was minimal expression in the corneal stroma, sclera, and iris [9]. Mutations in *LTBP2* have been associated with the Weill-Marchesani syndrome and the Weill-Marchesani-like syndrome, which are genetically heterogeneous disorders and are also caused by variants in fibrillin-1 and *ADAMTS10* [10,11,12]. To our knowledge, this is the first published case of a JOAG subject associated with recessively inherited variants of *LTBP2*.

## 2. Material and Methods

### 2.1. Subject and Clinical Evaluation

The current study has been approved by Institutional Review Board Committees at the University of Maryland School of Medicine, Baltimore, MD (HP00064793). All methods used in the study followed the precepts of the Declaration of Helsinki. Informed written consents were obtained from all investigated individuals prior to inclusion in the study. Detailed interviews were conducted with family members to gather information on pedigree structure, comorbidities, onset of disease, and initial symptoms. Visual acuity was assessed by using the standard Snellen chart. Intraocular pressure was measured by using Goldmann applanation tonometry considered to be the gold standard when measuring intraocular pressure [13]. Visual Field testing was completed with a standard automated perimetry with the Humphrey Visual Field (Zeiss Meditech, Jena, Germany). Optical coherence tomography (OCT) of the retinal nerve fiber layer was completed by using Heidelberg Spectralis OCT 2 (Heidelberg Engineering, Heidelberg, Germany). Fundoscopy and slit lamp bio-microscopy were also performed. Peripheral blood samples were collected from all the participants for DNA extraction.

### 2.2. Whole Exome Sequencing and Bioinformatic Analyses

Whole exome sequencing (WES) was used to identify the disease-associated variants. For WES, the genomic library of the proband was recovered for exome enrichment by using the Agilent SureSelect Human Expanded All Exon V5 (62 Mb) kit and sequenced on an Illumina HiSeq2500 with average 100X coverage. Data analysis used the Broad Institute’s Genome Analysis Toolkit [14]. Reads were aligned with the Illumina Chastity Filter with the Burrows Wheeler Aligner [15]. Variant sites were called using the GATK UnifiedGenotyper module. Single nucleotide variant calls were filtered by using the variant quality score recalibration method [14]. Filtration of candidate variants was performed as described previously [16]. Sanger sequencing was used to confirm the segregation of identified variants in the family. Clustal omega (https://www.ebi.ac.uk/Tools/msa/clustalo/) multiple sequence alignment was used to appraise the evolutionary conservation of the identified variants. Multiple pathogenicity prediction programs e.g., SIFT [17], Polyphen2 [18], MutationTaster [19], MutationAssessor [20], Fathmm [21], Provean [22], and CADD [23] were used to examine the impact of identified variants.

## 3. Results

### 3.1. Clinical Findings

**Figure 1:**
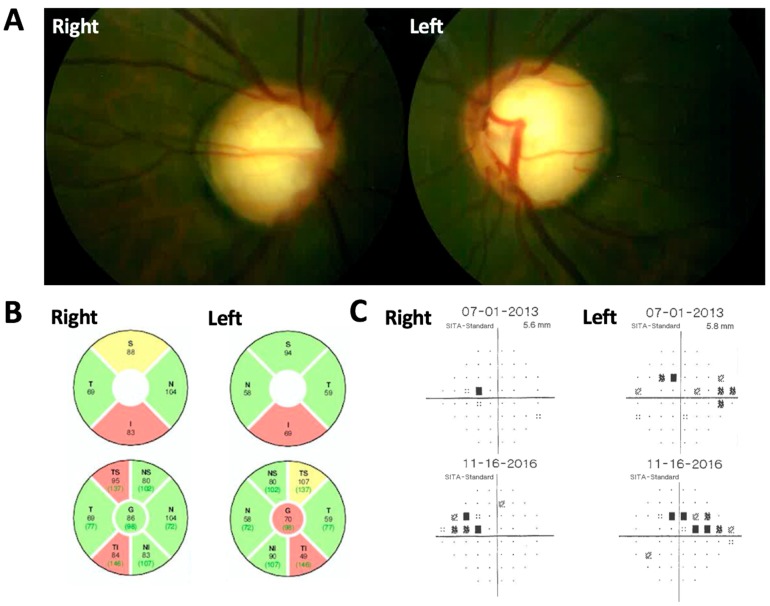
Proband ocular assessments. (**A**) Fundus photo of right and left eyes. (**B**) OCT-RNFL (ocular coherence tomography retinal nerve fiber layer) of both eyes. Key: Superior (S), Temporal (T), Nasal (N), Inferior (I). (**C**) Right and left nasal step visual field defects consistent with glaucomatous damage.

The proband was a male of Indian origin with past ocular history significant only for high myopia who was diagnosed with JOAG at age 20 with bilateral intraocular pressures (IOP) of 18 and 20 mm Hg and subsequent development of glaucomatous visual field (VF) defects. He was, subsequently, closely followed for several years. He was referred to clinical genetics and also agreed to participate in a genetics study to identify any associated genetic mutation. His karyotyping revealed normal chromosomal counts (46, XY). There was no known history of consanguinity and he was the product of an uncomplicated pregnancy. Psychomotor development was normal and he reached developmental milestones at proper ages. There were also no signs of any physical abnormalities. Best-corrected visual acuity was 20/20 in each eye with a correction of −6.50 in both eyes. Intraocular pressure was between 18 mm Hg in the right eye and 19 mm Hg in the left eye upon the presentation while on tafluprost. The ophthalmologic exam showed a normal corneal diameter of 12 mm horizontally, central corneal thickness of 597 microns in the right eye and 563 microns in the left eye, absence of Haab’s Striae, bilaterally clear lens with no signs of cataract, open angles on gonioscopy, a cup to disk ratio of 0.9 in both eyes (Figure 1A), and an unremarkable retinal exam. The ocular coherence tomography retinal nerve fiber layer (OCT-RNFL) exam showed superior and inferior never fiber layer thinning in both eyes (Figure 1B) and VF showed nasal defects in the left eye. These findings were consistent with glaucomatous optic neuropathy. The proband was ultimately put on maximum tolerated medical therapy for glaucoma (Brinzolamide/Brimonidine Tartrate in both eyes three times daily, Bimatoprost 0.01% in both eyes once daily) given concern for VF progression over a three-year period (Figure 1C). The patient has no other significant medical conditions. Both parents were also examined. VF and OCT showed that both parents were suspect for glaucoma. However, there is, otherwise, no known family history of glaucoma. IOP of the father was 18 mm Hg in the right eye and 19 mm Hg in the left eye. The mother’s IOP was recorded as 14 mm Hg in both eyes. The proband is an only child.

### 3.2. Mutations Detection in Latent Transforming Growth Factor-Beta-Binding Protein 2

**Figure 2:**
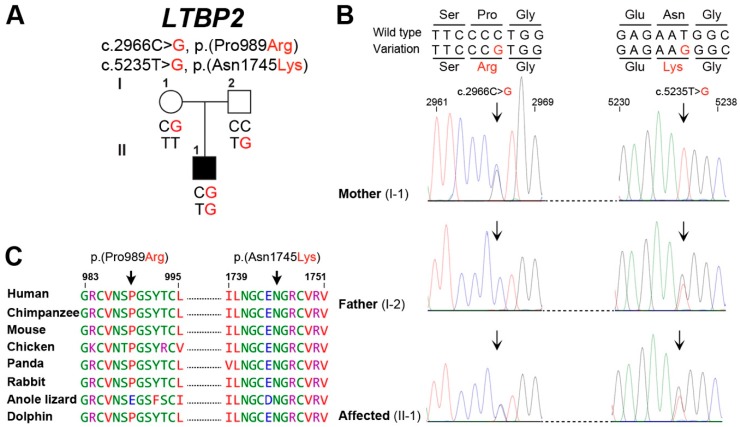
Compound heterozygous variants in latent transforming growth factor-beta-binding protein 2 (*LTBP2*) are associated with juvenile open angle glaucoma (JOAG). (**A**) Family with JOAG showing segregation of two variants of *LTBP2*. Affected individual is shown by filled symbol. (**B**) Shown also are the Sanger sequencing DNA chromatograms of *LTBP2* for the normal (parents) and affected individuals. The mutated nucleotides are marked with arrows. (**C**) Amino acids conservation in orthologous species for the p.(Pro989Arg) and p.(Asn1745Lys) variants. The wild type residues (p.Pro989 and p.Asn1745) are represented with arrows.

Bioinformatics analysis of whole exome sequencing data generated using the DNA sample of proband (II: 01) revealed two compound heterozygous variants [c.2966C>G, p.(Pro989Arg); c.5235T>G, p.(Asn1745Lys)] in *LTBP2*, which segregates with the phenotype in a recessive pattern (Figure 2A). The maternally inherited *LTBP2* variant (c.2966C>G) [p.(Pro989Arg)] is found in ExAC and 1000 genome with allele frequency as high as 0.005512 and 0.0056, respectively, but has not been previously associated with glaucoma and is absent in the ClinVar database [24]. The second variant (c.5235T>G), which is inherited from the father, has very low frequency in ExAC (2.231 × 10^−4^) and 1000 genome databases (2.0 × 10^−4^) and was absent from the in house exome database (Table 1). 

Both of the identified missense variants [p.(Pro989Arg), p.(Asn1745Lys)] are predicted to replace evolutionary and highly conserved residues (Figure 2B) and are predicted to be deleterious by multiple in silico prediction algorithms including Polyphen2, SIFT, MutationTaster, MutationAssessor, Fathmm, and Provean and had high levels with CADD scores (Table 1). We also used the HOPE [25] prediction program to further assess the effects of these two missense variants on the secondary structure of the encoded protein. The p.Pro989 is located in EGF (Epidermal Growth Factor)-like domain 7 of the encoded protein, which is important for calcium binding and subsequent biological function [26]. Due to the differences in charge, size, and hydrophobicity, the proline replacement with arginine at amino acid position 989 is predicted to result in ligands repulsion as well as disturb the protein local secondary structure by the loss of hydrophobic interactions in either the core of the protein or the surface of the protein. Similarly, the p.(Asn1745Lys) missense variant could result in the repulsion of similar charged ligands (Ca2+) and disrupt the protein function.

## 4. Discussion

LTBP2 has been associated with primary congenital glaucoma, pseudoexfoliation glaucoma, and POAG in older individuals [27]. We present the first case of JOAG in the United States associated with compound heterozygous variants of *LTBP2*. Parents of the proband were not related and happened to be carriers of two distinct variants of *LTBP2*. The proband is atypical in his clinical presentation. His intraocular pressure was never measured above 21 mmHg and he had documented the progression of his glaucoma with intraocular pressures in the mid-teens. This is unusual for juvenile glaucoma, which is almost always associated with high intraocular pressures [4] with the exception of a few reported cases [28,29]. The presence of VF deficits especially given the degree of optic nerve excavation that this patient has is associated with a loss at least of 20% to 30% of retinal ganglion cells [30]. This patient has typical findings of advanced glaucoma including a large cup to disc ratio and progressive VF deficits. He also has significant myopia, which is a risk factor for glaucoma [31].

LTBP2 is an extracellular matrix microfibrillar protein with 20 EGF-like domains containing calcium-binding sites to carry out protein-protein interactions and is involved in sustaining the ciliary muscle trend as well as normal development of the eye anterior chamber [8]. *LTBP2* variants have been found to cause the Weill-Marchesani syndrome, which is associated with glaucoma and severe myopia [10]. As of August 2018, 26 disease-causing variants in *LTBP2* have been identified according to the Human Gene Mutation Database (HGMD). Functional studies have suggested that variants in *LTBP2* affects both the trabecular meshwork as well as scleral collagen and may obstruct the fluid outflow and results in increased IOP and glaucoma [9]. Since the patient did not have high IOPs, it is more likely that the variants have a more deleterious functional effect on the sclera than the trabecular meshwork. Greater elasticity of scleral collagen may be associated with greater biomechanical strain on the lamina cribrosa and ultimately glaucomatous optic neuropathy [32]. It’s intriguing that, in humans, different alleles of *LTBP2* cause phenotypes at different developmental stages and with variable severities. One possibility is that the genetic background modifies the phenotypic outcome of *LTBP2* mutations. Alternatively, differences in mutant alleles of *LTBP2* may directly account for the different phenotypic outcomes. Knock-in alleles of mouse LTBP2 engineered to model human variants could aid in addressing these questions. Our clinical and molecular analysis of *LTBP2* alleles may prove useful for a future genetic diagnosis and counseling as well as for molecular epidemiology studies of JOAG.

## Figures and Tables

**Table 1:** Compound heterozygous variants of *LTBP2* causing JOAG.

Gene	*LTBP2*	*LTBP2*
hg19 Position	chr14:74978010	chr14:74968229
Genomic region	14q24.3	
Reference genomic allele	G	A
Alternate genomic allele	C	C
GenBank	NM_000428.2	
cDNA change	c.2966C>G	c.5235T>G
Amino acid change	p.(Pro989Arg)	p.(Asn1745Lys)
Segregates with the phenotype	Yes	Yes
dbSNP rsID	rs76172717	rs528254230
ExAC allele frequency	0.005512	0.0002231
No. ExAC European (Non-Finnish) alleles	2 homozygotes	0 homozygotes
ExAC Europeans (Non-Finnish) MAF	0.002054	0.000015
1000 genome	0.0056	0.0002
TOPMed	0.0015	0.00002
ClinVar	Absent	Absent
MAF in-house exomes (n = 109)	0.00917	Absent
SIFT	Damaging	Damaging
Polyphen2	Possibly damaging	Probably damaging
MutationTaster	Damaging	Damaging
MutationAssessor	Medium	Medium
Fathmm	Damaging	Damaging
Provean	Deleterious	Deleterious
CADD	25.1	24.3

LTBP2: Latent transforming growth factor-beta-binding protein 2; JOAG: Juvenile open angle glaucoma.
